# Characterization of Pore Size Distribution and Water Transport of UHPC Using Low-Field NMR and MIP

**DOI:** 10.3390/ma16072781

**Published:** 2023-03-30

**Authors:** Xin-Rui Xiong, Jun-Yan Wang, An-Ming She, Jian-Mao Lin

**Affiliations:** 1 School of Materials Science and Engineering, Tongji University, Shanghai 201804, China; 2 Key Laboratory of Advanced Civil Engineering Materials, Ministry of Education, Tongji University, Shanghai 201804, China; 3 Fujian Expressway Science and Technology Innovation Research Institute Co., Ltd., Fuzhou 350001, China

**Keywords:** low field nuclear magnetic resonance (LF-NMR), water absorption, pore size distribution, limestone powder (LS), steam curing

## Abstract

Water transport is vital for the durability of ultra-high performance concrete (UHPC) in engineering, but its absorption behavior requires further comprehension. This study investigates the impact of silica fume (SF) and metakaolin (MK) on water absorption in UHPC matrix with a high volume of limestone powder (LS) under two curing temperatures, and the variation in water transport with pore size obtained by low field nuclear magnetic resonance (LF-NMR). Relations between cumulative water absorption with other properties were discussed, and the pore size distribution (PSD) measured by Mercury intrusion porosimetry (MIP) was compared with that determined by LF-NMR. Results showed that MK outperformed SF in reducing water absorption in UHPC matrix, containing 30% LS under steam curing due to the synergistic effect between MK and LS. The incorporation of LS greatly affected the water absorption process of UHPC matrix. In samples without LS, capillary and gel pores absorbed water rapidly within the first 6 h and slowly from 6 h to 48 h simultaneously. However, in samples with 30% LS, gel pore water decreased during water absorption process due to the coarsening of gel pores. MK was able to suppress gel pore deterioration caused by the addition of a large amount of LS. Compared with PSD measured by MIP, NMR performed better in detecting micropores (<10 nm).

## 1. Introduction

Ultra-high performance concrete (UHPC) is a new cement-based composite material with ultra-high strength and outstanding durability [1]. The high strength is achieved by the use of a large amount of cement, which produces high hydration heat and greenhouse gas emissions [2]. To address this issue, inert powders, such as limestone powder (LS), have been employed to replace cement [3]. However, the accompanying negative impacts on the mechanical properties and durability caused by the high proportion of inert particles cannot be disregarded [4,5] and limits the widespread application of UHPC.

There are two common approaches applied for this challenge: adding the supplementary cementitious materials (SCMs) and increasing the curing temperature [6,7]. The hydration of SCMs, such as silica fume (SF) and metakaolin (MK) can be accelerated by steam curing, resulting in additional hydration products and denser matrix, thus favoring mechanical properties and durability [8]. UHPC doped with MK has higher strength under steam curing than that doped with SF [9], and the synergistic effect between MK and LS can further mitigate the negative impact of high-volume LS on UHPC performance [10,11]. However, existing studies only illustrated the positive effects of MK on mechanical properties of steam-cured UHPC. The influence of steam curing on water transport in UHPC containing a large amount of LS with MK remains unclear. Water absorption is a crucial property of concrete as it enables aggressive ions to penetrate and damage its durability [12,13]. Thus, it is essential to have a better comprehension of the water absorption behavior of concrete.

Current research on water absorption in concrete mainly focuses on two areas: common basic characteristics (i.e., water absorption content and rate) and underlying mechanisms of water absorption performance, and moisture transport and its relation to microstructure. It has been revealed that various factors can influence the porosity and pore size of cement-based materials, which consequently affects water absorption performance [14,15,16,17]. Thereinto, small capillary pores (10–100 nm) perform a major role in water absorption process [18,19]. As for moisture transport and its relation to microstructure, recent studies have begun experimenting with low field nuclear magnetic resonance (LF-NMR) [20,21,22].

LN-NMR is an instrumental noninvasive technique for characterizing water transport of cement samples. It takes pore water as probe with the signal amplitude proportional to pore water content and relaxation time showing a linear correlation with pore size. [23]. Several studies have used LF-NMR to analyze moisture transport during drying of concrete, demonstrating its advantages in continuous and non-destructive monitoring of moisture transport [24,25,26]. However, research on the application of LF-NMR to characterize the entire process of water absorption is fairly limited and even exhibits contradictory conclusions. Xiong et al. [27] studied the moisture transport patterns of an external thermal insulation composite system and found that pores in the range of 0.01 μm to 1 μm reached saturation within 4 h of water absorption, while pores larger than 1 μm started to absorb water in later stages. However, Zhang et al. [26] observed a different result in white cement, where large pores were filled first followed by small pores, and gel pores (<10 nm) continued to absorb water even after capillary pores became saturated due to gel swelling. This suggests that water absorption behaviors vary greatly with different cement-based materials. Previous studies have mostly used concrete with a high water-binder ratio (>0.3), so the water transport mechanism described earlier may not be applicable for UHPC (with a low water-binder ratio < 0.2). Furthermore, the effect of mineral admixtures and curing temperature on water transport of UHPC is still unclear. Therefore, systematic experimental and analytical studies on the water transport of UHPC are essential for a better understanding of water absorption behavior of UHPC and its durability.

The main aim of this study is to investigate the effect of SF and MK on water absorption rate of UHPC matrix with high-volume limestone powder under steam curing and standard conditions and reveal the water transport pattern with LF-NMR. First, the effects of types and content of mineral admixtures and curing temperature on compressive strength, water absorption, and pore structure tested by Mercury intrusion porosimetry (MIP) of UHPC matrix are systematically demonstrated and discussed. Afterwards, the water intrusion process and the evolution of water-bearing pore distribution during water absorption process were characterized using LF-NMR, and two water absorption patterns were proposed. Finally, the relationship between water absorption, compressive strength, and porosity was discussed, and the pore size distribution (PSD) measured by MIP and LF-NMR was compared. This study aims to highlight the benefits of using LF-NMR for monitoring water absorption processes, offer new insights into the water absorption behavior of UHPC matrix and provide guidance for enhancing its durability.

## 2. Materials and Methods

### 2.1. Materials

**Figure 1:**
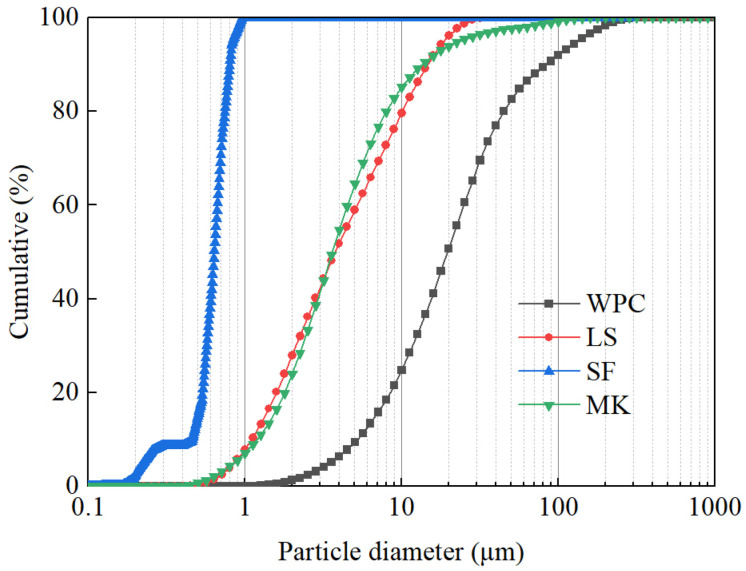
Particle size distributions of materials used in this study.

White Portland cement (WPC), supplied by Jiangxi Yinshan White Cement Company (Yinshan, China) and conforming to the Chinese standard GB/T 2015–2017 [28], was used to reduce the influence of paramagnetic substances on LF-NMR testing. The limestone powder (LS), silica fume (SF), and metakaolin (MK) were supplied by local vendors. Their chemical compositions measured by X-ray fluorescence (XRF) are given in Table 1, and their particle size distributions determined by laser granulometer are presented in Figure 1. Two kinds of quartz sands were utilized as aggregates. Sand 1 had particles size between 0.212 mm and 0.380 mm, and sand 2 between 0.380 mm and 0.830 mm. Superplasticizer used was polycarboxylate superplasticizer. The water used in this work was tap water.

### 2.2. Mixture Design and Mixing Procedure

Eight mixes under two curing conditions were prepared. Table 2 shows the mix proportioning details. In all mixes, water accounted for 23% of the total volume of mortar, while sand accounted for 37%. LS content was 30% of the volume of the binder content. The water to binder ratio (w/b) was changing between 0.19–0.20 by adjusting the amount of superplasticizer (SP) to maintain the fluidity of fresh mortar from 330 mm to 350 mm, conforming to the Chinese standard GB/T 2419-2005 [29]. SF and MK were added with different volumes ranging from 5% to 15%. The mixture coded L0 means 100% white cement for the cementitious material system. L is the content of LS. S is the content of SF, and M is the content of MK. For example, L30S10 represents 60% volume of cement, 30% volume of LS, and 10% volume of SF for the cementitious material system.

### 2.3. Specimen Preparation and Curing

All powders were added into a planetary-type mixer for dry mixing at a low speed for 30 s. Then, water was added and stirred at a low speed for about 5 min. After that, all sands were added and stirred at a low speed for 2 min, then at a high speed for 30 s. The specimens were kept in molds for 24 h at room temperature and humidity ambient. After demolding, the specimens were cured under two different curing treatments:

Treatment I: standard curing (20 ± 1 °C, RH ≥ 98%) until the time of testing.

Treatment II: steam curing for 2 days at 90 °C followed by standard curing until the time of testing.

### 2.4. Experimental Methods

#### 2.4.1. X-ray Fluorescence (XRF) and Laser Granulometer

Chemical composition was analyzed by XRF with PANalytical Axios (PANalytical, The Netherlands). Loss on Ignition (LOI) was determined gravimetrically by weighing pre-dried materials before and after ignition at 1050 °C. The weight loss includes CO_2_ and H_2_O. The particle size distributions were determined by laser granulometer using OMEC TopSizer (OMEC, China).

#### 2.4.2. Compressive Strength

The compressive strength at 3 days, 7 days, and 28 days of samples was measured in accordance with the Chinese standard GB/T 17671-2021 [30]. Three prisms with size of 40 × 40 × 160 mm^3^ for each mixture were used for flexural strength test. Then, six half-cut specimens obtained after flexural strength test were used for compressive strength test. The test results were obtained by averaging six measured compressive strength values. The loading rate used for compressive strength test was 2.4 kN/s.

#### 2.4.3. Water Absorption Test

The water absorption test was performed on cubes of 50 mm at the age of 28 days. Before measuring, first, samples were desiccated at 40 °C in a drying oven until the relative of mass change was less than 1% for 5 days. Then, the initial mass (*M*_0_) of every sample after cooling was recorded. Samples were completely submerged in a water tank with water surface 5 cm higher than the top surface of the cubes. Subsequently, the cubes were taken out and the surface moisture was wiped off with a wet towel to get the saturated weight (*M*_t_) at different time intervals (0 min, 1 min, 5 min, 10 min, 20 min, 30 min, 1 h, 2 h, 3 h, 4 h, 5 h, 6 h, 12 h, 24 h, and 48 h, respectively). The cumulative water absorption *(CWA*, %) can be calculated by the formula as follows:

(1) $$CWA=\frac{M_{t}-M_{0}}{M_{0}}\times 100\%$$

#### 2.4.4. Mercury Intrusion Porosimetry (MIP)

Porosity measurements were conducted by MIP with AutoPore V9600 (MicroActive, USA) at age of 28 days. Fragment samples (around 1 cm^3^) were selected from the center part of specimens after strength test, then immersed in ethanol (reagent grade, 98 purity) for 3 days to stop the hydration. Afterwards, they were dried in a drying oven at 40 °C for 1 day to remove the remaining ethanol. The measurement range of pore size is 0.003–120 μm.

#### 2.4.5. Low Field Nuclear Magnetic Resonance (LF-NMR)

The hydrogen protons in waters confined in pores in cement paste can be detected by different transverse relaxation time (*T*_2_). The difference in water content and water-bearing pore location will affect signal intensity and *T*_2_. *T*_2_ is affected by three fluid relaxation mechanisms, bulk relaxation *T*_2B_, surface relaxation *T*_2S_, and diffusion relaxation *T*_2D_, which can be expressed as Equation (2):

(2) $$\frac{1}{T_{2}}=\frac{1}{T_{2B}}+\frac{1}{T_{2S}}+\frac{1}{T_{2D}}$$

The Relaxometry theory put forward by Brownstein [31] explains that the transverse relaxation process of water in micropores is dominated by surface-limited relaxation [32]. Thus, *T*_2B_ and *T*_2D_ can be ignored. Based on the fast exchange theory, a theoretical expression for the correlation between *T*_2_ and the pore size can be expressed by Equation (3) [23,32]:

(3) $$\frac{1}{T_{2}}\approx \frac{1}{T_{2S}}=\rho_{2}\left(\frac{S}{V}\right)=\rho_{2}\frac{2F_{s}}{d}$$

where *d* is the pore diameter; *S/V* is the specific surface area of pores; *F*_s_ represents the pore shape factor, which is assumed as 2 for cylindrical pores; and *ρ*_2_ is the surface relaxivity. Therefore, NMR *T*_2_ spectrum can be translated into a PSD curve [33,34]:

(4) $$d=4\cdot \rho_{2}\cdot T_{2}$$

The LF-NMR spectrometer produced by Niumag Electric Corporation (Micro-MR20) (Niumag, China) was used to monitor the evolution of water-bearing pores at the micro level during water absorption in this study. The temperature of magnet and probe assembly was held constant at 32 ± 0.01 °C. The Carr–Purcell–Meiboom–Gill (CPMG) sequence was applied for measuring the *T*_2_ spectrum. Table 3 shows the main parameters. The specimen for the NMR test was a small cylinder with a diameter of 25 mm and a height of 35 mm. The samples at 28 days of age were submerged in a water tank and tested at the same time intervals as the water absorption test during water absorption process.

## 3. Results and Discussion

### 3.1. Compressive Strength

**Figure 2:**
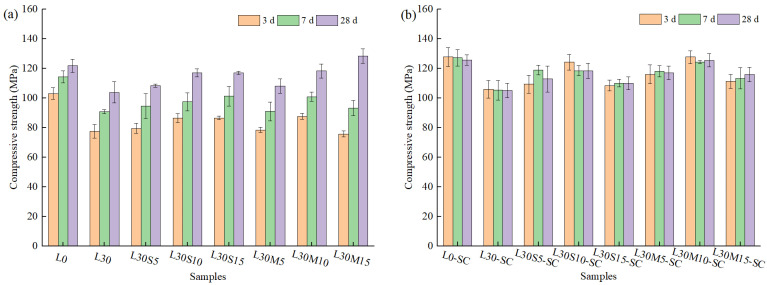
Compressive strength of samples at time of 3 d, 7 d, and 28 d: (**a**) Standard curing; (**b**) Steam curing.

The compressive strength for all the samples at 3 days, 7 days, and 28 days with two different curing conditions is shown in Figure 2. It can be found that the strength of all groups continuously increased with curing time under standard curing conditions, which was consistent with the growth tendency of the literature [35]. Compared with the L0 group, the compressive strength of L30 group at 3 days and 28 days decreased by 24.7% and 14.7%, respectively. This is due to the dilution effect of limestone powder [5]. Generally, the compressive strength increased with SF and MK volume. This is attributed to the filling effect and high pozzolanic activity of SF and MK [36,37,38,39,40,41].

For steam-cured samples, the compressive strength at 3 days was equivalent to that of standard-cured samples at 28 days, indicating that steam curing accelerated the hydration process and improved early compressive strength. However, a slight compressive strength reduction was observed in nearly all steam-cured samples at 7 days or 28 days. This result corresponds with previous researches on UHPC matrix containing diverse mineral admixtures [42,43,44], but deviates from normal concrete, which exhibited consistent growth with age after steam curing [45]. This phenomenon may be attributed to the low water-binder ratio of UHPC. High temperature accelerates subsequent hydration reactions and solid-phase expansion. The compact structure of low water-cement ratio cementitious material restricts the expansion of hydration products, leading to the generation of more cracks in the matrix and, consequently, a slight decrease in compressive strength [46]. This result is also consistent with the findings of the pore structure analysis discussed in Section 3.3 with more macropores in steam-cured samples.

In addition, the compressive strength of L30S15-SC and LS30M15-SC was lower than that blended with 5% and 10%, with the compressive strength at 28 days age even lower than that of the standard-cured specimens at the same age. This is due to the poor homogeneity of UHPC fresh paste owing to the excessive incorporation of 15% SF and MK, which had a high requirement for water [44,47], as evidenced by the increased SP dosage in Table 2, and the nonuniform distribution of hydration products caused by a high curing temperature [45]. Thus, excessive incorporation of 15% SF and MK can lead to a decline in compressive strength. The higher compressive strength was achieved 118.2 MPa and 125.4 Mpa at 28 days for L30S10-SC and L30M10-SC, respectively.

### 3.2. Water Absorption

**Figure 3:**
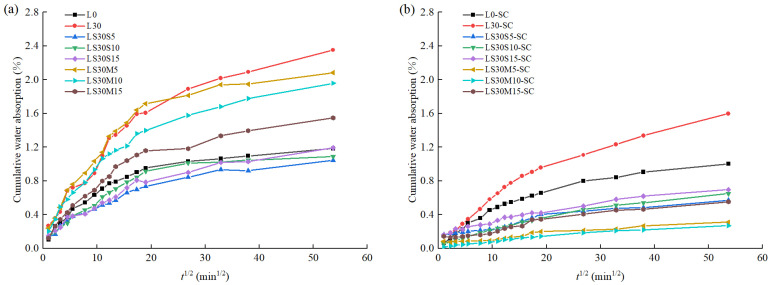
Cumulative water absorption against square root of immersion time: (**a**) Standard curing; (**b**) Steam curing.

Figure 3 shows the cumulative water absorption (*CWA*) of the samples. *CWA* of all samples under both conditions increased with immersing time, and it increased rapidly in the first 6 h, then slowly continued to increase, which was discovered in many literature [14,48]. Under standard curing condition, L30 represented the highest *CWA* for 2.352% at 48 h on account of the dilution effect of high volume of LS, which reduced hydration products [22]. Samples containing SF exhibited a lower *CWA* in comparison to samples containing MK, which was in line with the results discovered by Tafraoui [9]. Incorporation of 5% SF reached the lowest *CWA* for 1.044% at 48 h soaking time, i.e., 53.66 sqr (time/min), 1.25 times lower than that of L30.

For steam-cured samples, the water absorption of each sample was lower than its corresponding sample under standard curing, implying that steam curing can remarkably densify the matrix and reduce the water absorption. The accelerated hydration process during steam curing led to the production of more hydration products, resulting in refined pore structures [49], as shown in Section 3.3. Moreover, the water absorption rate of samples mixed with MK was lower than that with SF, which was contrary to the standard curing situation, reflecting that MK is more effective in decreasing water absorption than SF when steam curing is applied. This is because of the high pozzolanic reactivity of MK and a stronger synergistic effect between MK and LS under steam curing [50].

It is worth noting that incorporation of 10% MK resulted in the lowest *CWA* of 0.271% at 48 h of soaking time, which was 4.92 times lower than that of LS30-SC. This CWA of 0.271% is 3–5 times lower than that of UHPC matrix incorporated with nano-silica reported by Mohammed [51], and 1.5 times lower than the CWA for 0.421% of UHPC matrix at 24 h of soaking time [9]. Additionally, it is thus more than 10 times lower than those of an ordinary concrete, as 9% [26].

### 3.3. Pore structure

**Figure 4:**
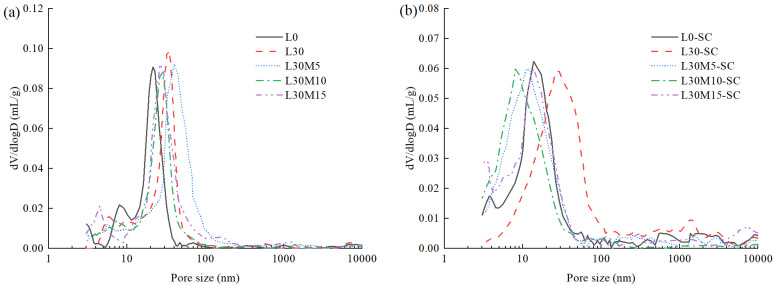
Pore structures of UHPC matrix with different MK replacement levels: (**a**) Standard curing; (**b**) Steam curing.

Figure 4 shows the pore structures of UHPC matrix with different MK replacement volumes. The pore size was mainly in the range of 3–100 nm. The most probable pore size of L0, L30, L30M5, L30M10, and L30M15 under standard curing were 21.45 nm, 34.46 nm, 39.8 nm, 25.57 nm, and 27.94 nm, respectively, and the corresponding groups under steam curing were 13.76 nm, 26.29 nm, 11.57 nm, 8.04 nm, and 13.60 nm, respectively, which can be concluded that steam curing can obviously refine the capillary pore size. The pore refinement effect of 30% LS group was not obvious, while that of the group mixed with LS and MK was the largest. This is probably because reaction between LS and MK generates more additional Ca-aluminates hydration products under steam curing condition to fill the pores and refine the pore structure [37,52]. This is the main reason for the significant reduction in water absorption of cement-limestone-metakaolin ternary system.

Interestingly, the steam-cured samples had a higher proportion of macropores than the standard-cured samples. However, the higher proportion macropores did not contribute to an increase in water absorption according to the results of water absorption test in Section 3.2. This phenomenon will be further investigated in Section 3.4, where the curves of microcrack or void water during water absorption remained almost unchanged will be presented.

### 3.4. Water Transport Process and Evolution of Water-Bearing Pores

**Figure 5:**
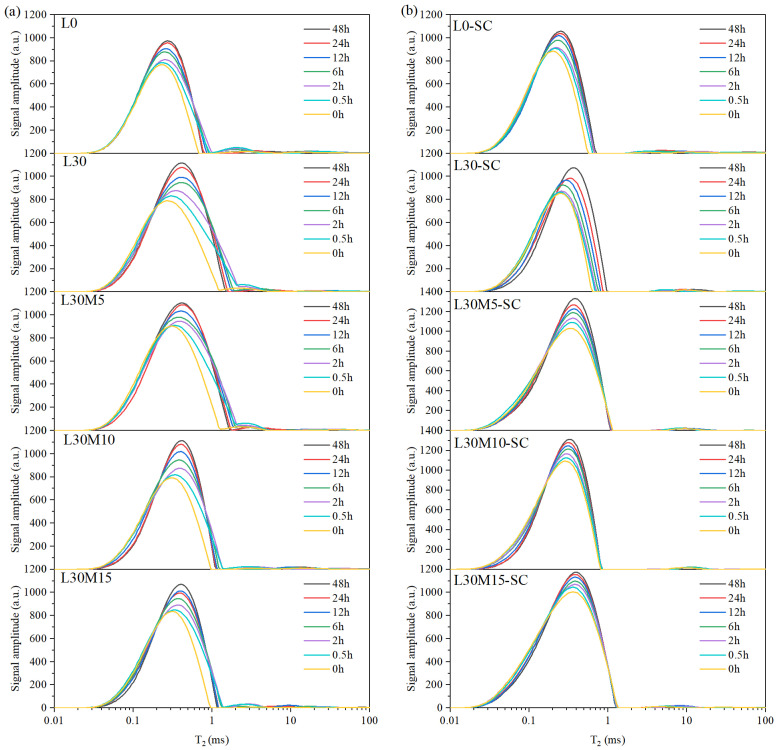
Evolution of *T*_2_ spectra for specimens after different time of water absorption: (**a**) Standard curing; (**b**) Steam curing.

**Figure 6:**
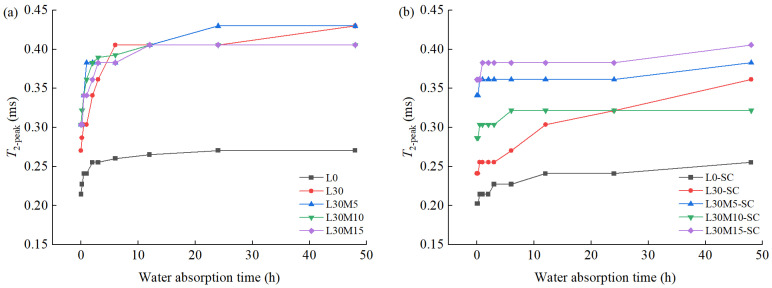
Evolution of *T*_2-peak_ during water absorption: (**a**) Standard curing; (**b**) Steam curing.

*T*_2_ spectra collected at 0 h, 0.5 h, 2 h, 6 h,12 h, 24 h, and 48 h during water absorption for specimens L0, L30, L30M5, L30M10, L30M15, L0-SC, L30-SC, L30M5-SC, L30M10-SC, and L30M15-SC were presented in Figure 5. The spectra of standard-cured samples in Figure 4a displayed a bimodal or trimodal distribution including a main peak and 1 or 2 small peaks, while steam-cured groups in Figure 4b had a main peak. The abscissa value corresponding to the main peak vertex is defined as *T*_2-peak_. Evolution of *T*_2-peak_ during the water absorption for each specimen is plotted in Figure 6.

The main peak of standard-cured samples spanned 0.01 ms to 2 ms, whereas the peak of steam-cured samples ranged from 0.01 ms to 1 ms, indicating that water was mainly distributed in smaller pores after steam curing. For the movement of the main peak during water absorption, all specimens showed the same trend. Within the first 6 h, the main peak grew higher, and *T*_2-peak_ shifted to the right, indicating a rise in water content and a shift towards a larger pore of water. After 6 h, the main peak and *T*_2-peak_ increased slowly until it reached a stable level.

**Figure 7:**
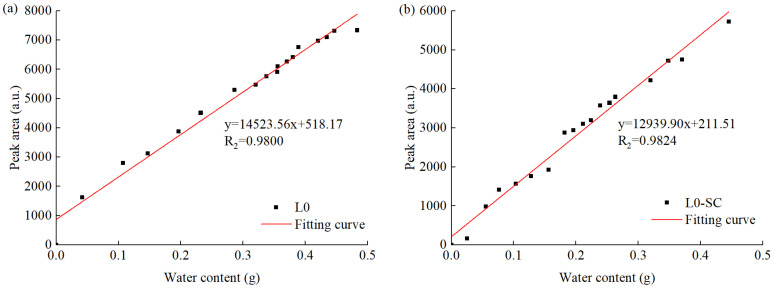
Relationship between absorbed water content and peak area (L0 and L0-SC were applied as an example): (**a**) L0; (**b**) L0-SC.

A good linear correlation with R^2^ > 0.98 was found by fitting the water absorption content and total *T*_2_ peak area of samples is presented in Figure 7. It validates the reliability of LF-NMR in monitoring water content based on the change of peak area during water absorption, which has been verified in the literature [53]. According to reference [54], water with *T*_2_ values below 0.28 ms is located in gel pores, water with *T*_2_ values ranging from 0.28–3 ms is found in capillary pores, and water with *T*_2_ values greater than 3 ms is present in microcracks or air voids.

**Figure 8:**
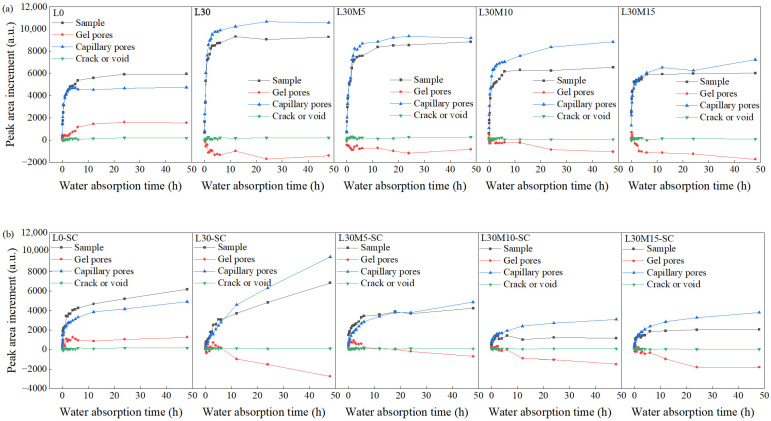
Peak area increments of different pores in water absorption process: (**a**) Standard curing; (**b**) Steam curing.

Figure 8 depicted the peak area increment, subtracting the peak area of dry samples, of different pores during water absorption process, encompassing water in the specimen, gel pores, capillary pores, and microcracks or air voids. Evidently, for all specimens regardless of curing condition, the change of capillary water followed the same trend as the total water in the specimen. The results were in accordance with the literature [26]. The signal variation of water in gel pores differed for L0 and L0-SC group compared to the other groups containing 30% LS, as gel pores water slowly increased for L0 and L0-SC (same as [26]) mainly due to the characteristics of gel swelling [26,55,56] while decreasing for the latter. The reduction in gel pores was directly related to the addition of LS, implying that the addition of LS resulted in pore coarsening of the UHPC matrix slightly during water absorption. The reduction rate of gel pores at 48 h water absorption time for L30, L30M5, L30M10, and L30M15 were 14.00%, 5.73%, 9.26%, and 10.61%, respectively, and for L30-SC, L30M5-SC, L30M10-SC, and L30M15-SC were 18.29%, 3.91%, 8.40%, and 9.59%, respectively, which indicated that MK could mitigate this degradation under both curing conditions. This may be attributed to the aluminum phase present in metakaolin, which bridges C-S-H and makes the hydration products and UHPC matrix denser [57]. Additionally, the micro-aggregate and filling effects of metakaolin particles further refine the pore structure [58,59]. In addition, the signal of water in the microcracks or air voids remained stable throughout the water absorption process. A similar result was obtained in earlier findings [27]. This can be explained by the fact that the larger the pore size, the lower the capillary suction stress, and the less the amount of absorbed water [27].

From the perspective of mixed proportions, the incorporation of LS led to an increase in the peak area increment of capillary pore water, while the addition of MK resulted in a decrease. Moreover, capillary pore water in steam-cured samples was lower than that in standard-cured ones. These findings aligned with the results of the water absorption test in Section 3.2. Notably, for L30-SC, the peak area increments of capillary pore water kept increasing, while gel pore water kept decreasing throughout the entire water absorption process. This trend persisted even after 6 h, while the curves of other samples tended to flatten. Additionally, the maximum *T*_2_ continuously shifted to the right shown in Figure 4b similar to the phenomenon obtained in the literature [60]. These results indicated the water instability of L30-SC, which may negatively affect the durability of the UHPC [60]. Additionally, L30M10-SC and L30M15-SC had the lowest peak area increment of water in capillary pores and in the specimen, indicating the densest microstructure.

Therefore, two water absorption patterns for UHPC matrix with and without LS were observed: (1) For samples without LS (L0 and L0-SC), capillary pores and gel pores absorb water rapidly in the first 6 h and slowly from 6 to 48 h simultaneously, while microcracks and air voids did not contribute to the water absorption. (2) For samples with 30% LS, capillary pores rapidly absorb water in the first 6 h and slowly from 6 h to 48 h. The gel pores coarsen gradually during the water absorption process, resulting in a decrease in water in gel pores. Although steam curing and reactive mineral admixtures affect the absorbed water content and water absorption rate, they have minimal impact on the water absorption pattern.

### 3.5. Relationship between Cumulative Water Absorption and Other Properties

**Figure 9:**
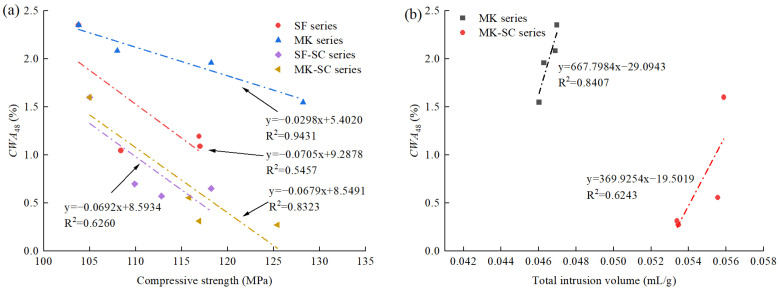

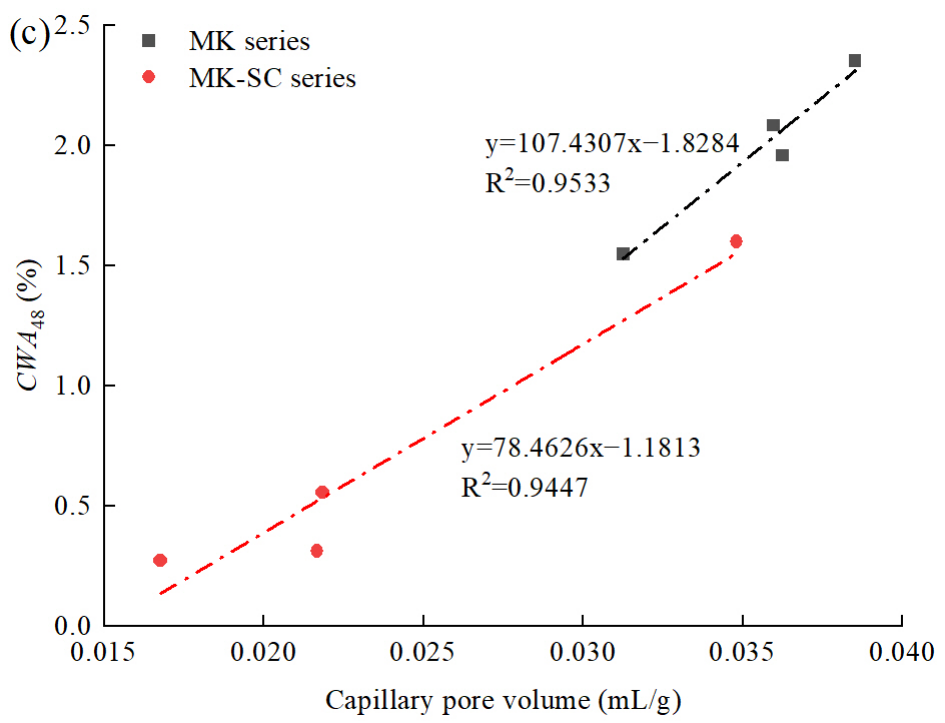
Relationship between cumulative water absorption at 48 h and other properties: (**a**) Compressive strength; (**b**) Total intrusion volume; (**c**) Capillary pore volume.

Figure 9 shows the relationship between cumulative water absorption at 48 h (*CWA*_48_) and other properties. The samples with similar mix proportions and the same curing conditions were grouped into series, such as the SF series (L30, L30S5, L30S10, and L30S15) and SF-SC series (L30-SC, L30S5-SC, L30S10-SC, and L30S15-SC).

Figure 9a shows the relationship between compressive strength and *CWA*_48_. *CWA*_48_ decreased with increasing compressive strength, as reported in the literature [16,61]. MK and MK-SC series had a higher R^2^ than SF and SF-SC series, indicating the type of SCMs that had an effect on the relationship. Additionally, *CWA*_48_ steam-cured samples were smaller than that of standard-cured samples at the same strength value.

Figure 9b,c depict the relationship between the total intrusion volume and capillary pore volume (10–200 nm) tested by MIP with respect to *CWA*_48_. Figure 9b shows that a good linear relationship was found between the total intrusion volume and *CWA*_48_ for MK series (R^2^ = 0.8407), but a weaker correlation for MK-SC series (R^2^ = 0.6243). Despite possessing a greater total intrusion volume than MK series, the MK-SC series exhibited a lower *CWA*_48_, thereby indicating that UHPC matrix with a higher total pore content may not necessarily exhibit higher water absorption capability. In other words, the total pore volume does not singularly determine the water absorption performance, which is different from normal concrete with a high water-binder ratio (>0.3) [48,56].

Figure 9c reveals a strong linear correlation between *CWA*_48_ and capillary pore volume in both series, regardless of the curing condition, thereby suggesting that capillary pores significantly contributed to water absorption. Notably, steam-cured samples with a higher total pore volume exhibited a lower capillary pore volume, resulting in a reduced water absorption rate, which further confirmed the dominant role of capillary pore volume over total pore volume in water absorption.

### 3.6. Comparisons of PSD Measured by MIP and LF-NMR

**Figure 10:**
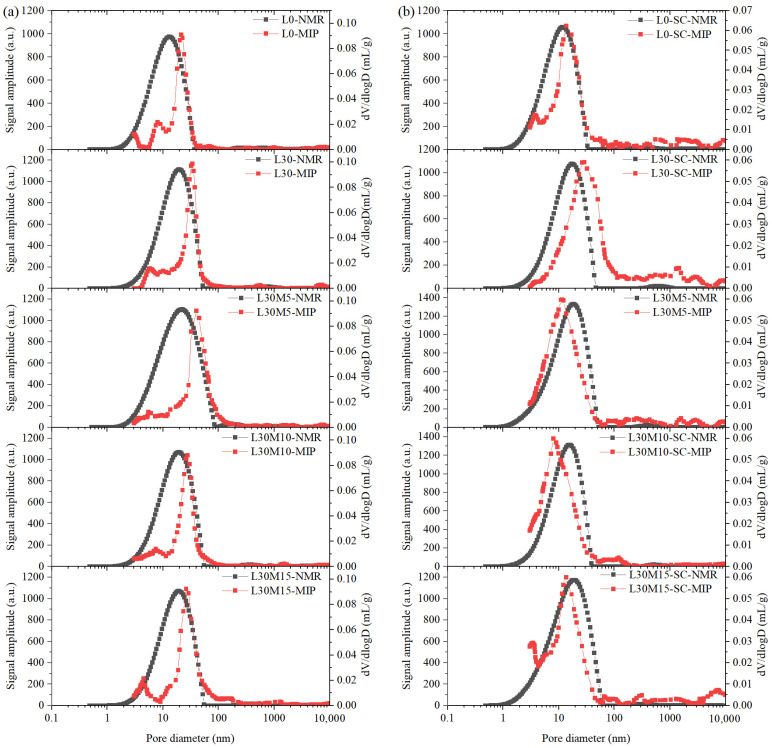
Comparisons of the PSD measured by LF-NMR and MIP: (**a**) Standard curing; (**b**) Steam curing.

Surface relaxation (*ρ*_2_) is taken as 12 nm/ms for this work [62,63]. As the peak area changes of most samples became flat in the later stages, the 48 h *T*_2_ spectrum was selected to convert to PSD curve [62]. The literature [64] showed that the *T*_2_ spectrum of the sample at 24 h of spontaneous water imbibition was similar to that after vacuum saturation, so the sample with 48 h of self-absorption can provide relatively accurate pore distribution information. The *T*_2_ spectrum at 48 h of water absorption was translated into a PSD curve and compared with the MIP PSD in Figure 10. The main peak of PSD curves measured by MIP matched NMR’s curves on the right side, but it was lower on the left side, except for steam-cured samples containing MK. High mercury injection pressure used in MIP can cause damage to microstructure, and thus, limit detection of small pores [65].

**Figure 11:**
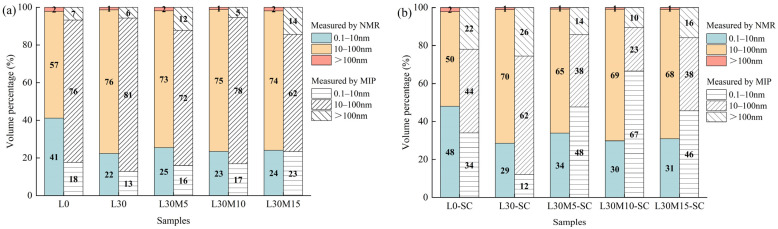
The volume percentage for different types of pores measured by LF-NMR and MIP: (**a**) Standard curing; (**b**) Steam curing.

To quantitatively compare the PSD curves measured by two methods, the pores were divided into three categories: micropores (<10 nm), mesopores (10–100 nm), and macropores (>100 nm) [65]. Figure 11 displays the volume percentage of each pore group measured by MIP and LF-NMR. MIP detected fewer micropores than NMR, except for steam-cured samples containing MK, of which MIP exhibited a higher proportion for pores smaller than 10 nm compared to LF-NMR. This phenomenon was also observed by literature [62]. This may be because MIP measures the entry size of pores rather than the real size, and the “ink-bottle” effect will cause an overestimation of small pores [66,67,68]. For macropores, MIP detected a higher proportion of macropores than LF-NMR, irrespective of the curing condition and mineral admixtures. Two reasons can be put forward. First, MIP testing damaged the specimen, resulting in an increase in the proportion of macropores. Second, the short waiting repetition time (TW) employed in the LF-NMR test is inadequate for capturing water in larger pores [62].

To conclude, it is not possible to achieve a perfect match between the pore distributions obtained from MIP and LF-NMR because of the working mechanism. NMR exhibits better performance in detecting small pores, whereas MIP is more effective in detecting larger pores. To obtain a comprehensive understanding of pore distribution, both methods can be used in combination, depending on the specific materials being studied.

## 4. Conclusions

The effect of silica fume and metakaolin under standard curing and steam curing on the water absorption behavior of UHPC matrix by LF-NMR and MIP were studied. The conclusions are drawn as follows:

1. An amount of 30% volume of limestone powder showed a significant decrease in compressive strength and increase in water absorption regardless of curing temperature. Silica fume and metakaolin improved the compressive strength and lowered the water-absorptivity. For water absorption reduction, metakaolin performed better than silica fume under steam curing condition due to the synergistic effect with limestone powder.
2. Steam curing obviously decreased the most probable pore size. The pore refinement effect of 30% LS group (L30) was not significant (reduced from 34.46 nm to 26.29 nm), while the group mixed with 10% MK (L30M10) had the most significant reduction from 27.94 nm to 8.04 nm. Although steam-cured samples had a higher proportion of macropores than the standard-cured samples, this did not contribute an obvious increase in water absorption.
3. LF-NMR is applicable for monitoring the water absorption process, and the water absorption patterns for the UHPC matrix vary with the presence of LS. In samples without LS (L0 and L0-SC), capillary pores and gel pores rapidly absorbed water within the first 6 h and slowly from 6 to 48 h at the same time, while microcracks and air voids contributed minimally to water absorption. In samples with 30% LS, capillary pores followed a similar trend to samples without LS, but gel pores coarsened gradually with the water absorption process, resulting in a decrease in gel pore water. MK mitigated this degradation under both curing conditions. The water absorption pattern is minimally affected by steam curing and reactive mineral admixtures, despite their impact on cumulative water absorption.
4. The cumulative water absorption decreased with increasing compressive strength in general. The cumulative water absorption of steam-cured samples was smaller than that of standard-cured samples at the same strength value. Moreover, the linear correlation between capillary pore volume and water absorption was better than the total pore volume, which indicates capillary pores perform a dominant role in water absorption over total pore volume.
5. *T*_2_ spectrum collected after 48 h of water absorption can be adopted to estimate pore distribution in UHPC matrix and compared with PSD measured by MIP. LF-NMR is more adept at identifying micropores, whereas MIP excels at detecting larger pores.

## Figures and Tables

**Table 1:** Chemical compositions and physical properties of WPC, LS, SF, and MK.

	WPC	LS	SF	MK
Chemical compositions (wt%)				
Al_2_O_3_	3.28	0.23	1.31	51.10
SiO_2_	20.69	0.63	85.21	47.06
Fe_2_O_3_	0.26	0.20	0.61	0.32
CaO	67.84	52.45	0.06	0.08
MgO	1.90	2.84	0.04	0.06
Na_2_O	0.37	-	6.40	0.04
K_2_O	0.49	-	0.29	0.09
SO_3_	4.58	0.05	0.21	0.01
LOI	1.50	43.21	5.08	1.02
Physical properties				
Density (kg/m^3^)	3036	2699	2138	2742

**Table 2:** Mixture proportions of UHPC mortar (kg/m^3^).

Mix ID	Cement	LS	SF	MK	Sand 1	Sand 2	Water	SP	Defoamer
L0 *	1 210	0	0	0	294	686	227	6.5	2
L30 *	847	323	0	0	294	686	227	4.0	2
L30S5 *	786	323	42	0	294	686	227	2.7	2
L30S10 *	726	323	85	0	294	686	227	3.0	2
L30S15 *	665	323	128	0	294	686	227	3.2	2
L30M5 *	786	323	0	54	294	686	227	3.0	2
L30M10 *	726	323	0	109	294	686	227	3.2	2
L30M15 *	665	323	0	164	294	686	227	3.4	2

* The specimen was cured under standard curing and steam curing both. The mix ID of steam-cured specimens is followed by the suffix “SC”, which will be mentioned in the following content.

**Table 3:** Sequence parameters for LF-NMR testing.

Parameters	Values
Spectrometer frequency (SF)	12 MHz
Offset of frequency(O1)	349,956.95 Hz
90° Pulse Length (P1)	5.2 μs
180° Pulse Length (P2)	9.8 μs
Waiting time for repeated (TW)	400,000 ms
Accumulated sampling times (NS)	32
Number of 180° pulses (NECH)	400
Echo time (TE)	0.18 ms

## Data Availability

The data presented in this study are available within the article.
